# Altered expression of ionotropic L-Glutamate receptors in aged sensory neurons of *Aplysia californica*

**DOI:** 10.1371/journal.pone.0217300

**Published:** 2019-05-23

**Authors:** Justin B. Greer, Edward M. Mager, Lynne A. Fieber

**Affiliations:** 1 Department of Marine Biology and Ecology, University of Miami Rosenstiel School of Marine and Atmospheric Science, Miami, FL, United States of America; 2 Department of Biological Sciences, University of North Texas, Denton, TX, United States of America; Bilkent University, TURKEY

## Abstract

The simplified nervous system of *Aplysia californica* (*Aplysia*) allows for detailed studies of physiological and molecular changes in small sets of neurons. Sensory neurons of the biting and tail withdrawal reflexes are glutamatergic and show reduced L-Glutamate current density in aged animals, making them a good candidate to study age-related changes in glutamatergic responses. To examine if changes in ionotropic L-Glu receptor (iGluR) transcription underlie reduced physiology, mRNA expression of iGluR was quantified in two sensory neuron clusters of two cohorts of *Aplysia* at both sexual maturity (~8 months) and advanced age (~12 months). Sensory neuron aging resulted in a significant overall decrease in expression of iGluR subunits in both sensory neuron clusters and cohorts. Although the individual subunits differentially expressed varied between sensory neuron clusters and different cohorts of animals, all differentially expressed subunits were downregulated, with no subunits showing significantly increased expression with age. Overall declines in transcript expression suggest that age-related declines in L-Glu responsiveness in *Aplysia* sensory neurons could be linked to overall declines in iGluR expression, rather than dysregulation of specific subunits. In both sensory neuron clusters tested the N-methyl-D-aspartate receptor subtype was expressed at significantly greater levels than other iGluR subtypes, suggesting an *in vivo* role for NMDAR-like receptors in *Aplysia* sensory neurons.

## Introduction

Aging is associated with impairments in neuronal function that result in declines in cognitive function and reflex systems of aged animals. A reduced number of synaptic contacts and reduced dendritic spine density can alter the strength of synaptic connections, leading to diminished excitatory postsynaptic potential amplitude in aged neurons [1–3]. The marine mollusk *Aplysia californica* (*Aplysia*) has been used extensively as a model for studying the effects of aging due to a relatively simple nervous system, easily identifiable neurons, and short life span of ~12 months. Well-mapped neural circuits in the *Aplysia* nervous system have allowed researchers to more closely correlate age-related changes in reflex behaviors with physiological and molecular changes in the relevant underlying neurons [4–9].

Throughout much of the animal kingdom, including *Aplysia*, the majority of fast synaptic transmission in the central nervous system is mediated by ionotropic glutamate receptors (iGluR) [10, 11]. Aging studies in vertebrates have suggested that reduced L-Glu current density can occur in the hippocampus and other brain regions of aged animals [12, 13]. In parallel with vertebrate studies, pleural ventral caudal (PVC) sensory neurons and buccal S cluster (BSC) sensory neurons in *Aplysia* are glutamatergic, and show reduced L-Glu current density and fewer neurons responding to L-Glu in animals of advanced age [14]. Although the role of glutamatergic responses in these neurons are unknown, reduced physiological responses with age may provide a model system for understanding the molecular basis of reduced L-Glu physiology in aged neurons. A previous whole-transcriptome study in our lab showed decreased expression of some iGluR subunits in aged PVC neurons, suggesting downregulation of iGluR may contribute to reduced physiology [15].

Vertebrate iGluR are classified into three subtypes based on selective agonists in vertebrates: N-methyl-D-aspartate (NMDA), α-Amino-3-hydroxy-5-methyl-4-isoxazolepropionic acid (AMPA), and kainate. Functional iGluR are formed as heterotetramers, consisting of iGluR subunits belonging to the same subtype. There is evidence that analogues of each of these vertebrate subtypes exists in *Aplysia*, with varying degrees of relatedness to their vertebrate counterparts [16]. The composition of *Aplysia* iGluR tetramers, if indeed its functional iGluR are formed as in vertebrates, is unknown.

NMDAR are the most well-conserved iGluR subtype across bilaterian species, including *Aplysia* [16], thus their function may be the most conserved across the bilaterian clades. NMDA elicits small amplitude excitatory currents in cultured *Aplysia* sensory neurons [17, 18] and application of the NMDAR antagonist 2-amino-5-phosphonopentanoic acid (APV) blocks only a minor component of L-Glu responses [19], making the contribution of NMDAR to L-Glu physiology unclear. NMDAR may be particularly important because they are highly permeable to Ca^2+^ [20], which activates second messenger cascades mediating neuronal plasticity [21]. AMPAR mediate the majority of fast excitatory transmission in the vertebrate nervous system and are much less conserved than NMDAR across bilaterian species. Although AMPA-induced currents were not present in *Aplysia* sensory neurons or in motoneurons of the abdominal ganglion [17, 22], it is expected that L-Glu is the endogenous neurotransmitter at these receptors. The role of kainate iGluR is less clear than NMDAR and AMPAR, and they are the most evolutionarily recent iGluR subtype. Kainate analogs elicited depolarizations and non-desensitizing inward currents in glutamatergic BSC neurons of *Aplysia*, similar to L-Glu-induced effects in these neurons [23]. Kainate is also an effective agonist in abdominal ganglion neurons [22].

To test the hypothesis that changes in iGluR transcription may underlie reduced iGluR current amplitude in aged sensory neurons, this study used qPCR to examine changes in iGluR transcription in mature and aged PVC and BSC sensory neurons in 2 cohorts of *Aplysia*. To provide context for vertebrate species, *Aplysia* iGluR subunits were classified as NMDAR or AMPAR based on homology with vertebrate subunits in a previous phylogenetics study [16].

Subunits with unclear phylogenetic relationships to vertebrate iGluR were not classified into a particular subtype.

## Materials and methods

### Husbandry

*Aplysia* from the University of Miami National Resource for Aplysia were reared from egg masses of wild-caught animals. Two cohorts were reared and maintained throughout their lifespan at a temperature of 13–15°C. Once the animals reached age 4 months they were held at 5 per cage to reach sexual maturity. Animals were fed an ad lib diet of *Agardhiella subulata* as previously described [7]. Mass was recorded each month for a random selection of 15 animals from each cohort beginning at age 3 months until the end of life, approximately age 12.5 months in both cohorts. Excess water was drained from the parapodial cavity prior to this measurement.

Sexual maturity for each cohort was designated as the first day an egg mass was laid. Reflex completion times were measured in animals at sexual maturity (M; age 8.5 months) and near the end of life (age 12 months) to quantify behavioral changes with age. Aged *Aplysia* have previously been defined as Aged I (AI) or Aged II (AII) based on progressively diminished reflexes and reductions in iGluR physiology of sensory neurons [14]. Times to complete TWR and righting were recorded in 8 animals from each cohort at each timepoint to determine the stage of aging for aged *Aplysia*. Mean reflex completion times were compared to Kempsell and Fieber (2014) and determined that age 12 month animals in these two cohorts were stage Aged II (AII).

### Righting

Righting was measured at M and then again at AII in 8 animals from each cohort per time point. An individual was placed in a 48x27x20 cm polycarbonate cage with flowing seawater and acclimated for five minutes before measurements of righting. The animal was picked up from the bottom of the cage and rolled off the palm of the hand at the top of the water column. Time was recorded from the moment the animal reached the cage bottom. It then righted itself and adhered to the bottom of the cage. The reflex was completed as it began to take its first crawling step. The measurement was recorded 3 times per individual, with a minimum of 5 minutes between trials, and the times averaged. Data were combined for all animals at each time point and a Student’s t-test was used to determine significant differences in righting between age groups.

### TWR

TWR was measured at M and AII in the same individuals that were analyzed for the righting reflex. After righting measurements, animals were allowed to rest for 5 minutes. The animal was then placed on its foot in the center of the cage, allowed to adhere to the bottom, and was lightly held in place to measure initial resting length. TWR was initiated by pressing a blunted 21-gauge needle to the tip of the animal’s tail for approximately 1 sec to initiate withdrawal of the tail. Time was recorded from the initiation of the reflex until the animal relaxed the tail approximately 30%. Retracted length was recorded to calculate percent tail withdrawn. Measurements of duration and amplitude of TWR were recorded 3 times, with a minimum of 5 minutes between trials, and the measurements averaged. A Student’s t-test was used to calculate differences in TWR time and fraction of body length withdrawn between age groups.

### RNA extraction of select ganglia for qPCR

RNA was extracted from a six-animal subset tested for TWR and righting from each cohort at M, and 6 from each cohort at AII. An animal was first anesthetized by injection of 1/6^th^ body weight (by volume) of isotonic MgCl_2_. The animal was euthanized by severing the connectives and removal of all nervous system ganglia. Ganglia were immediately rinsed in artificial seawater (ASW: 417 mM NaCl, 10 mM KCl, 10 mM CaCl_2_, 55 mM MgCl_2_, and 15 mM HEPES-NaOH, pH 7.6) after removal.

PVC neurons were separated from the remainder of the pleural ganglia and immediately placed in Qiagen lysis buffer, with both left and right PVC clusters pooled into a single sample. The same procedure was used to harvest BSC neurons from the buccal ganglia. Total RNA from PVC and BSC neurons was extracted with the Qiagen RNeasy Micro Kit following the manufacturer’s instructions. Genomic DNA contamination was removed during RNA extraction according to manufacturer’s instructions (Qiagen).

Quantity of RNA from all tissues was analyzed using a NanoDrop (Model ND-1000). One hundred ng of total RNA from each sample was reverse transcribed into cDNA using SuperScript III reverse transcriptase (Invitrogen) with random hexamer primers following the manufacturer’s suggestions. The resulting cDNA was diluted 1:5 with nuclease free water to provide a final concentration equivalent to 1 ng RNA/1 μl cDNA for qPCR.

### qPCR

qPCR reactions were executed on a Stratagene Mx3005P with SYBR Green master mix (Invitrogen). Each reaction consisted of 10 μl of SYBR Green master mix, 2 μl of cDNA and 0.2 μM of forward and reverse primers in a total volume of 20 μl. Thermal cycler conditions were as follows: 95°C for 10 minutes, followed by 40 cycles of 95°C for 15 sec, 58°C for 30 sec, 72°C for 30 sec. All 12 iGluR subunits and the splice variant of Grin1 previously identified were tested. Primer sequences and amplicon lengths for each transcript can be found in Table A in S1 Dataset.

For cohort 1, sensory neuron clusters from 6 animals at M and at AII were analyzed with qPCR to quantify changes in iGluR expression. Two technical replicates were performed for each biological replicate. Absolute copy number for each replicate was calculated using serial dilutions of a known quantity of plasmid containing the target amplicon. PCR products for each gene were gel purified with a Qiagen gel purification kit and transfected into electrocompetent *E coli* cells using a TOPO TA cloning kit (ThermoFisher). Plasmids were purified with a Qiagen mini-prep plasmid kit and quantified on a Nanodrop to determine plasmid concentration. Plasmids were linearized by restriction digest and serial dilutions prepared from 10^6^−100 plasmid copies that were run in triplicate on each plate to calculate a standard curve for absolute copy number calculation.

For cohort 2, PVC and BSC neuron clusters from 4 animals were tested at each time point. These samples were run in technical triplicate instead of duplicates as were run for cohort 1. In this cohort, we tested the expression of several of the most highly expressed iGluR subunits from Cohort 1: Grin1-1, Grin1-2, Grin2, GluR1, GluR5, and GluR7. For cohort 2 quantification, target transcripts were normalized to GAPDH using the Gene Expression’s C_T_ Difference (GED) formula [24], which is a modified version of the ΔΔC_T_ method [25] that accounts for the efficiency of each reaction. First, ΔC_T_ was calculated for the gene of interest (GOI) in each biological sample using the formula ΔC_T_ = C_T_ (GOI)–C_T_ (GOI:reference), where the reference is an independent sample not used for other analyses. The same reference sample used was for each plate and contained PVC neurons from a sexually mature animal. Relative expression ratio (rER) was then calculated using the rER formula described by Schefe and Lehmann [24]. qPCR efficiencies used in the rER formula were calculated using Real-time PCR miner [26]. Relative transcription of aged/mature for each transcript was then calculated (mean rER(aged) / mean rER(mature). Raw C_T_ values used for all qPCR analysis are available in S1 File. Individual subunits were classified into vertebrate subtypes according to their relationship to vertebrate iGluR as defined in a previous study [16].

### Statistical analyses

For cohort 1, differential expression of each gene was calculated using a two-way analysis of variance (ANOVA) on absolute copy numbers, followed by Tukey’s HSD post hoc test for each subunit. Differential expression in cohort 2 was determined using a two-way ANOVA on the calculated rER values (described above), followed by Tukey’s HSD post-hoc test. Significance was defined at p ≤ 0.05 following multiple testing correction, and all p-values presented throughout are following multiple testing correction.

## Results

### Aging indices

Declines in reflex behaviors occur in the months preceding death and coincide with the timing of reduced L-Glu current densities in aged *Aplysia*. Average mass declined beginning approximately two months prior to AII measurements in both cohorts (Fig A in S1 Dataset), consistent with the aging timeline of previous hatchery studies [7, 27]. Time to right increased significantly at AII in both cohorts (Fig A in S1 Dataset). TWR also increased significantly in AII animals compared to M in both cohorts (Fig A in S1 Dataset), while TWR amplitude, measured as the fraction of body length withdrawn in response to tail touch, decreased significantly (Fig A in S1 Dataset). The observed TWR ≥20.1 sec and righting ≥18.1 sec for age 12 month animals of both cohorts corresponded to AII based on published stages of aging in *Aplysia* [14].

### Expression of iGluR subunits with age

To determine whether reduced L-Glu current density is correlated with altered expression of iGluR subunits, expression of each subunit was compared in PVC neurons of M and AII animals. To further characterize the effects of aging on iGluR subtypes, individual subunits were categorized into NMDA (Grin1, Grin2) or AMPA (GluR1, GluR2, GluR3, GluR4, GluR5, and GluR8) vertebrate receptor subtypes based on a previous phylogenetic study [16]. Predicted *Aplysia* iGluR subunits without clearly defined relationships to vertebrate iGluR (GluR6, GluR7, KA1, and KA2) were not assigned a subtype designation.

**Fig 1:**
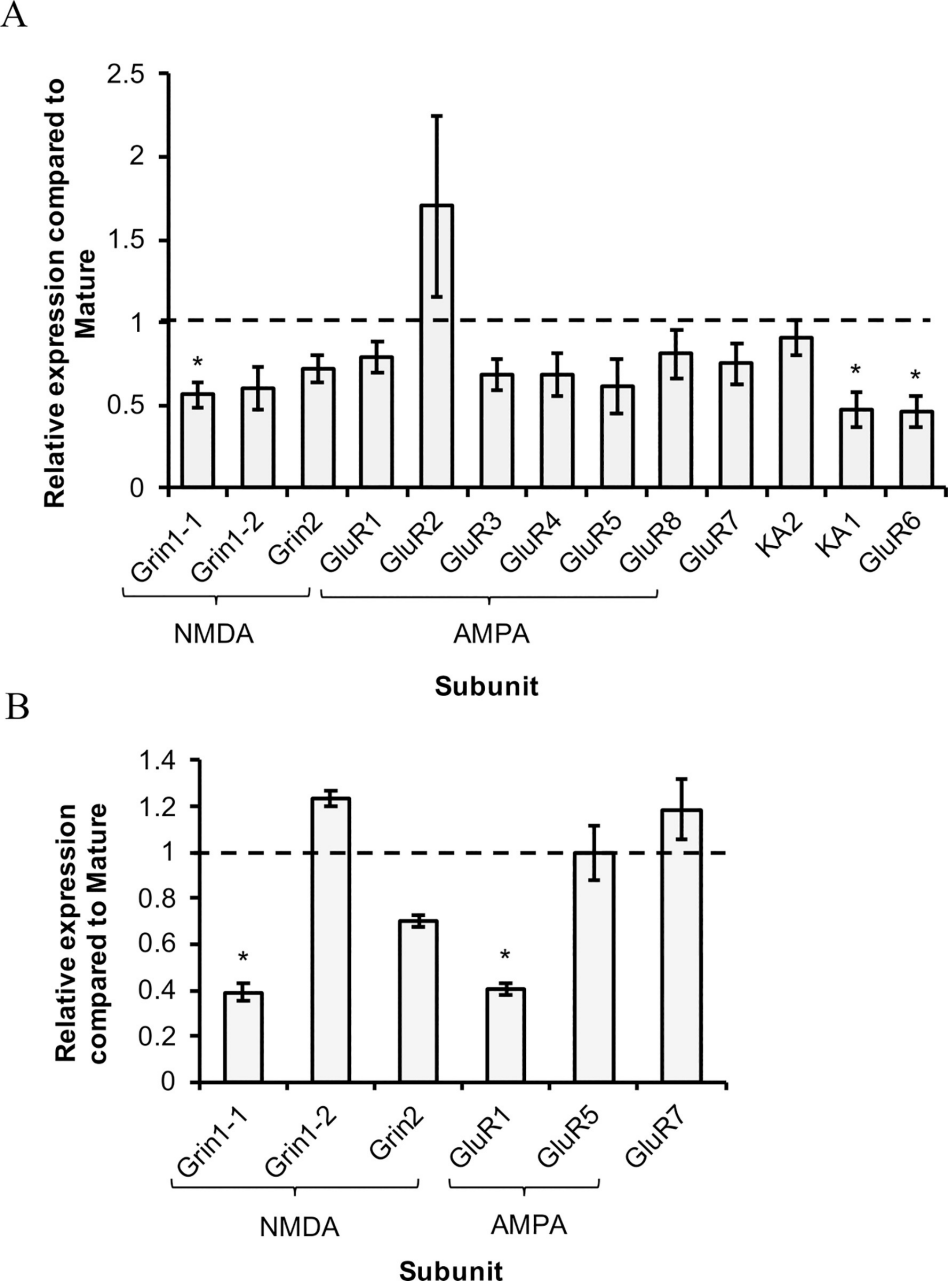
Relative expression of iGluR subunits in PVC sensory neurons with age. A) Relative expression for all iGluR subunits in AII compared to M (dotted line) in cohort 1. B) Relative expression of select iGluR subunits in cohort 2 AII compared to M (dotted line). All data are presented as mean relative expression ± SEM. *denotes significantly reduced expression compared to M (p≤0.05, two-way ANOVA, Tukey’s post hoc; n = 6 for cohort 1, n = 4 for cohort 2).

In PVC neurons of cohort 1 there was a statistically significant effect of aging on overall iGluR expression (*F*_1,12_ = 14.53, *p* < 0.05), as well as downregulation of 3 individual subunits in aged animals: Grin1-1, KA1, and GluR6 (Fig 1A). Several other subunits displayed >30% reduction in transcript expression with age but were not statistically different. In contrast, there were no subunits with significantly increased expression in AII animals compared to M. A subset of transcripts analyzed for cohort 1 were selected for analysis in cohort 2 to confirm the general expression pattern observed in the former (Fig 1B). Overall iGluR transcription was significantly decreased (*F*_1,5_ = 4.52, *p* < 0.05) and GluR1 and Grin1-1 subunits were significantly downregulated in aged neurons of cohort 2. Similar to cohort 1, significantly increased expression was not observed for any iGluR subunits.

**Fig 2:**
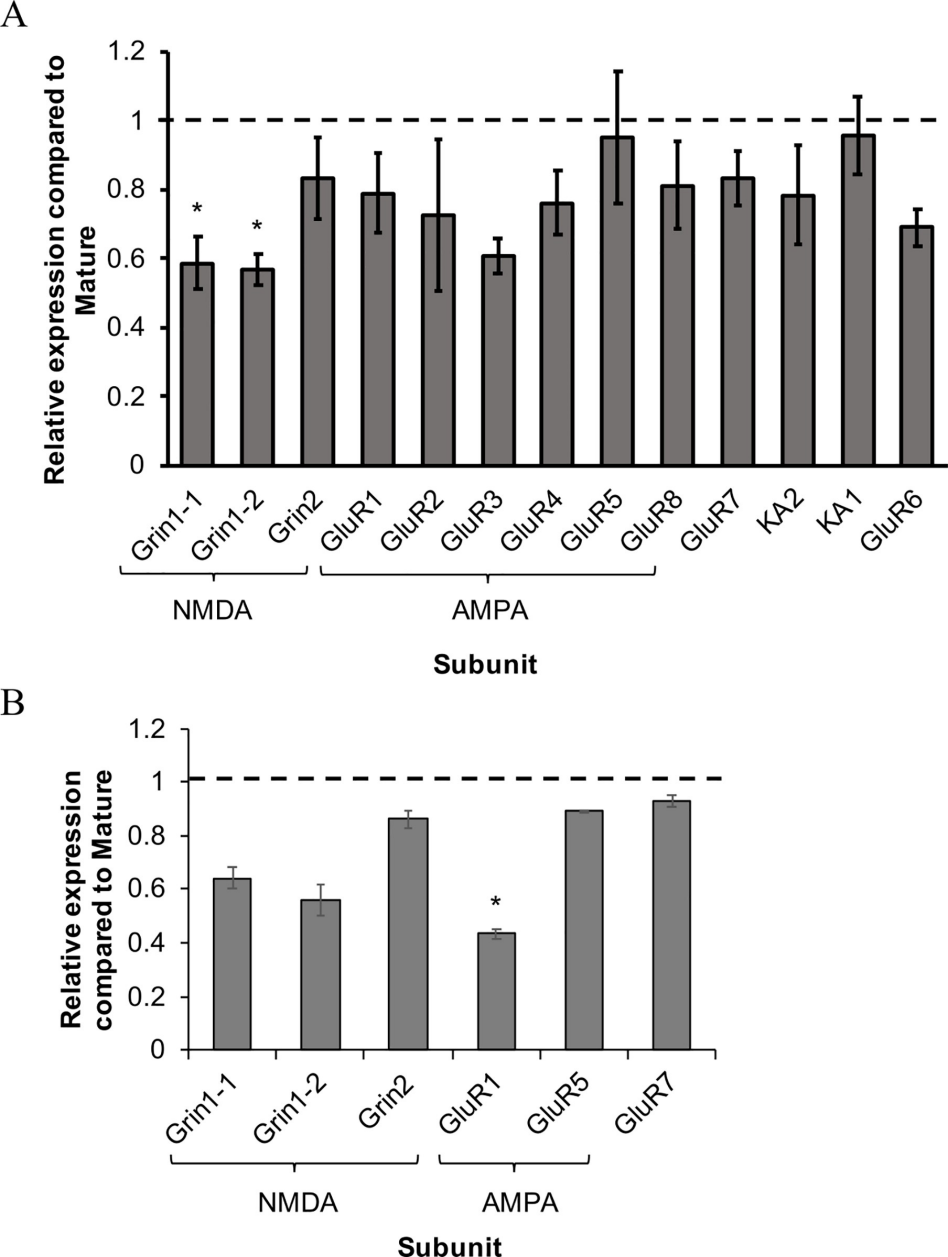
Relative expression of iGluR subunits in BSC sensory neurons with age. A) Relative expression for all iGluR genes in AII BSC sensory neurons compared to M (dotted line) in cohort 1. B) Relative expression for select subunits iGluR subunits in AII BSC sensory neurons compared to M (dotted line) in cohort 2. All data are presented as mean relative expression ± SEM. *denotes significantly reduced expression compared to M (p≤0.05, two-way ANOVA, Tukey’s post hoc; n = 6 for cohort 1, n = 4 for cohort 2).

BSC sensory neurons are also characterized by reduced L-Glu current density in AII animals [14] and were tested for transcriptional changes with age. In cohort 1 aging resulted in a significant decrease in overall iGluR transcription (*F*_1,12_ = 10.75, *p* < 0.05), and NMDA receptor subunits Grin1-1 and Grin1-2 were significantly downregulated in aged animals (Fig 2A). Overall reductions in iGluR transcription were also observed in BSC neurons of cohort 2 (*F*_*1*,*5*_ *=* 8.51, *p* ≤ 0.05). The AMPAR subunit GluR1 had significantly decreased expression in cohort 2 (Fig 2B).

### Differential expression of iGluR subunits between PVC and BSC neurons

**Fig 3:**
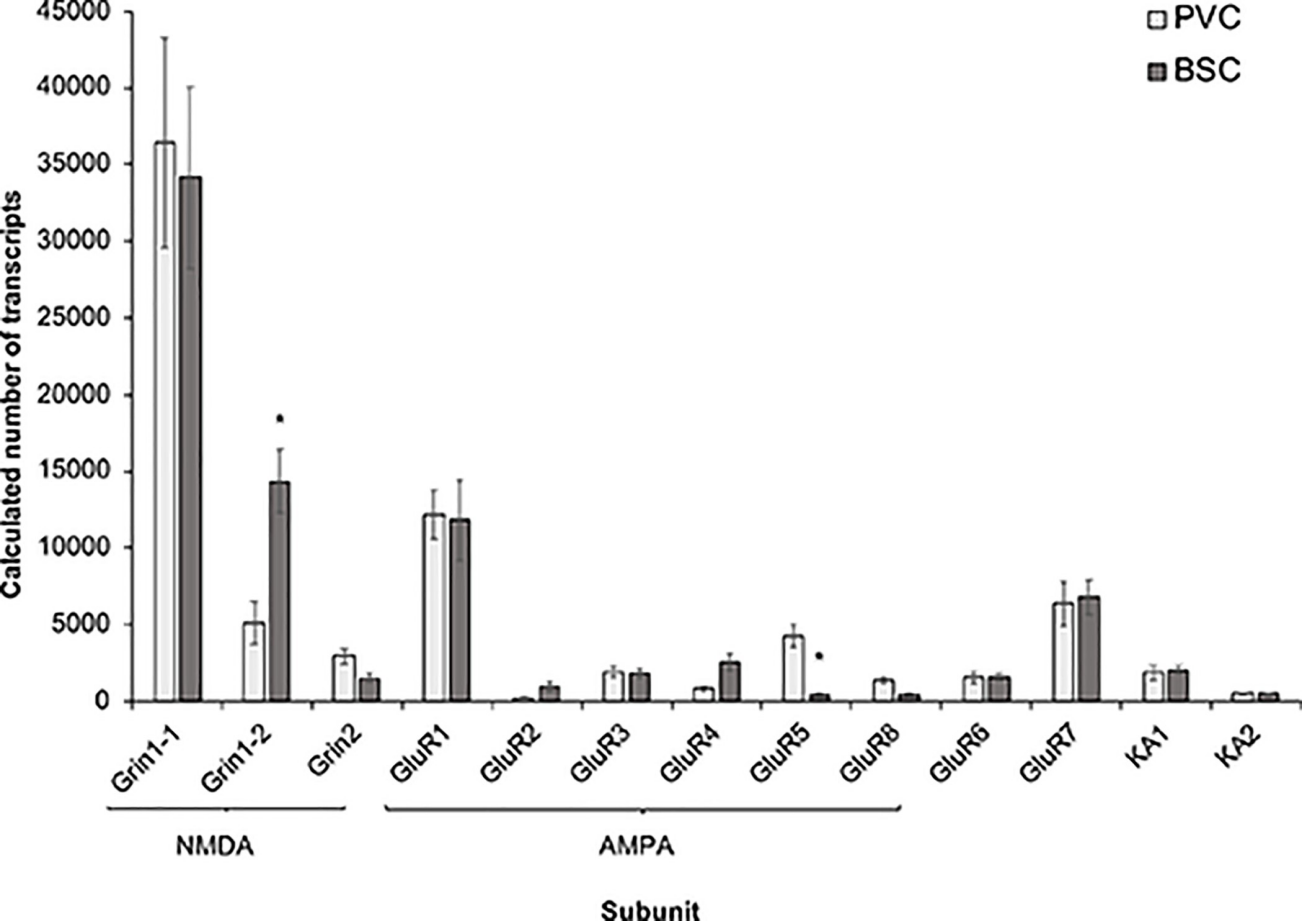
Comparative number of iGluR transcripts between PVC and BSC neurons. Number of transcripts is calculated per 100 ng of RNA from M animals only. *denotes significant differential expression (p≤0.05, Student’s t-test, Holm-Bonferroni correction).

To determine if subunit expression varies in different populations of sensory neurons, transcript expression of each iGluR subunit was compared between PVC and BSC neuronal clusters. Data were calculated using expression from M animals in cohort 1, since absolute copy number in this cohort allowed for direct comparisons of genes from different qPCR plates. Transcript expression of NMDAR subunit Grin1-2 was significantly greater in BSC neurons than PVC, and AMPAR subunit GluR5 expression was significantly higher in PVC neurons. Also apparent in Fig 3 is the high expression of Grin1-1, which was expressed at a minimum of >2 times the expression of other subunits in both PVC and BSC neurons (Fig 3). GluR1 was the most highly expressed AMPAR transcript in both neuronal clusters.

## Discussion

Previous studies in *Aplysia* have extensively documented age-related changes in behavior, neuronal function, neurotransmitter regulation, and transcriptional regulation [4, 28–31]. In this study we used the glutamatergic PVC and BSC sensory neurons clusters to examine molecular changes that may underlie declines in L-Glu physiology with age. Although the exact function of iGluR currents in sensory neurons of *Aplysia* is undemonstrated, PVC and BSC clusters provide a large number of easily identifiable and homogeneous neurons that may be a model for changes in glutamatergic transmission during learning or aging. PVC and BSC sensory neurons show diminished or absent iGluR during aging, suggesting that ion channel function may be compromised. We therefore examined transcriptional changes of iGluR subunits during aging as a potential contributor to reduced L-Glu physiology.

We observed a significant overall decrease in mRNA expression of iGluR subunits in advanced age neurons compared to the same neurons examined at the peak of maturity in PVC and BSC neurons of both cohorts. This may be important, because although an approximately equal number of genes are up and down regulated in aged *Aplysia* neurons [15, 29], downregulation of iGluR expression is specifically correlated with aging. This parallels studies in the cholinergic R15 bursting neuron of the abdominal ganglion, where alterations in acetylcholine-induced action potentials during aging corresponded with reduced expression of some acetylcholine receptors [5]. Further studies in R15 showed alterations in biological pathways for cell signaling, cellular function, and neurological diseases pathways also occurred and could contribute to reduced neurotransmission [29]. Age-related reductions in iGluR expression have been described in several vertebrate species [32–34] and reduced binding of L-Glu in aged neurons is linked to reduced numbers of functional iGluR [12].

In particular, the NMDAR subunit Grin1-1 was the most highly expressed iGluR subunit and exhibited >25% reduction in transcript expression in both clusters in both cohorts. Declines in mRNA and protein expression of Grin1 subunits have been described in the brain of aged rats, mice, and macaque monkeys, suggesting that Grin1 subunits are highly susceptible to the effects of aging [33–36]. In some instances the reductions in NMDAR protein expression may be greater than the observed reductions in mRNA [37]. Grin1 is expressed throughout the *Aplysia* nervous system [38], and is also widely expressed in *Lymnaea* neurons and mammalian brains [38–40], suggesting an *in vivo* role for NMDAR in iGluR physiology across Bilateria. However, the importance of reduced Grin1 expression to declines in L-Glu physiology of *Aplysia* are unclear. Vertebrate NMDAR are obligatory heterotetramers formed from two Grin1 and two Grin2 subunits [10, 41] and expression of *Aplysia* Grin1 in *Xenopus* oocytes does not induce L-Glu currents [38], suggesting that complete NMDAR in *Aplysia* are also likely heterotetramers of Grin1 and Grin2. Thus, because Grin2 is expressed at lower levels than Grin1, complete receptor assembly may be limited by Grin2 abundance. Grin2 was not differentially expressed in either PVC or BSC neurons of either cohort, and therefore the number of complete NMDAR could be unchanged by declines in Grin1 expression. Alternatively, Grin1 turnover rates may be much higher than Grin2, requiring greater transcription.

The effects of aging on expression of specific iGluR subunits varied between PVC and BSC neurons and in both cohorts. Inconsistencies in transcriptional changes during aging have previously been noted in both the *Aplysia* nervous system and the vertebrate brain [14, 29, 42, 43]. For example, NMDAR-mediated current density in rats and mice were reduced by 20–50% in some studies [13, 32, 44, 45], while NMDAR subunits were either upregulated or unchanged during aging in other studies [46, 47]. The inconsistencies in iGluR expression changes make it difficult to elucidate the mechanism underlying reduced L-Glu current density. Rather, as noted earlier, overall declines in iGluR expression suggest that the summation of reduced iGluR expression may contribute to declines in glutamatergic responses in aged *Aplysia* neurons. Decreased L-Glu-mediated transmission in the aging vertebrate brain is also attributed to altered composition of subunits comprising complete tetrameric AMPAR and NMDAR, conferring differences in biophysical and pharmacological properties of the receptors and conveying different messages from the same stimulus [41, 48]. Transcript levels are not sufficient to predict receptor composition, however, and further studies would be needed to address if changes iGluR composition contribute to physiological changes in *Aplysia*.

In summary, aging in *Aplysia* is a dynamic process that results in a suite of behavioral, physiological, and transcriptional changes. We observed a significant overall reduction in iGluR transcription in aged sensory neurons of the pleural and buccal ganglia compared to sexually mature animals. The specific subunits downregulated were inconsistent across tissues and cohorts, suggesting that diminished L-Glu responses could be the summation of reduced expression of many subunits, rather than alterations in specific subunits.

## Supporting information

S1 Dataset **Table A**. Primer sequences used for qPCR of iGluR subunits. **Fig A. Behavioral and morphological correlates of aging in two cohorts of *Aplysia***. Values expressed are mean±standard error. A) Animal mass estimated from wet weight decreased beginning approximately 2 months before AII measurements (n = 15). Dotted lines represent time points animals were sacrificed for qPCR. B) Time to right increased significantly in AII. C) Tail withdrawal time increased significantly in AII. D) Amplitude of tail withdrawal decreased significantly in AII, calculated as fraction of the initial length withdrawn.
(DOCX)

S1 File Raw Ct values for qPCR. (XLSX)
