# Supplementary material for: Altered expression of ionotropic L-Glutamate receptors in aged sensory neurons of Aplysia californica
Source: PLoS One. 2019 May 23;14(5):e0217300. doi: 10.1371/journal.pone.0217300 (PMC6532900; doi:10.1371/journal.pone.0217300)
Supplement: S1 Dataset — Table A. Primer sequences used for qPCR of iGluR subunits. Fig A. Behavioral and morphological correlates of aging in two cohorts of Aplysia. Values expressed are mean±standard error. A) Animal mass estimated from wet weight decreased beginning approximately 2 months before AII measurements (n = 15). Dotted lines represent time points animals were sacrificed for qPCR. B) Time to right increased significantly in AII. C) Tail withdrawal time increased significantly in AII. D) Amplitude of tail withdrawal decreased significantly in AII, calculated as fraction of the initial length withdrawn. (DOCX) [file pone.0217300.s001.docx]

Table A

| Gene | Primer Sequences for qPCR (5’-3’) | | Amplicon length (bp) |
| --- | --- | --- | --- |
| GluR1 | F  R | GCTTTGTGGACAACACCAGC  GGTTCTGCCATGATGATCGAC | 116 |
| GluR2 | F  R | GACTTAAAGGTGTCCAACGC  CGTCATACGACAAGCTCTTC | 145 |
| GluR3 | F  R | GAACCTTGACCCCAAGTTCTG  CCTATTCACGAGAGCTTTCG | 101 |
| GluR4 | F  R | CGTACTTGAGTTTGCGGTCC  GTGACCATATCGCTGCAGAC | 121 |
| GluR5 | F  R | CTTCACGGAGGAGTCAAAGTC  CTTCAGACGGATGCAGCACT | 146 |
| GluR6 | F  R | GAGATACGTGCTGGATCAGG  GGCTCATGATGGACTACAAC | 154 |
| GluR7 | F  R | CGAGACTGCTTTAGCCTACG  CCATTAGCCTGCCTTGTGAC | 100 |
| GluR8 | F  R | TCAAGTCGCTCAATCTCTCC  CCACGTCAACACACTTCTAGTC | 110 |
| KA1 | F  R | GCTACCGAAACCAGCTCAATC  GCAGTCACACGTACTTAAGAGG | 145 |
| KA2 | F  R | GATCTGGCTCTACGTCATTGG  CTTGTAACCCAGACACGGAC | 115 |
| Grin1-1 | F  R | GGGAAACATTGAGAAGC  GCTCCCAATGCAAACACAGC | 135 |
| Grin1-2 | F  R | GACACCAACGAAATAGACCTC  GCTCCCAATGCAAACACAGC | 146 |
| Grin2 | F  R | AGTTCACCTGCGACTCTGAC  CCGAGGTTCTCATCCTGAATC | 150 |

Fig A
